# A crystal structure of the human protein kinase Mps1 reveals an ordered conformation of the activation loop

**DOI:** 10.1002/prot.25651

**Published:** 2019-01-08

**Authors:** Jacomina C. Roorda, Robbie P. Joosten, Anastassis Perrakis, Yoshitaka Hiruma

**Affiliations:** ^1^ Division of Biochemistry Netherlands Cancer Institute Amsterdam The Netherlands

**Keywords:** activation loop, ATP, mitotic kinase, Mps1, spindle assembly checkpoint, TTK, X‐ray crystallography

## Abstract

Monopolar spindle 1 (Mps1) is a dual‐specificity protein kinase, orchestrating faithful chromosome segregation during mitosis. All reported structures of the Mps1 kinase adopt the hallmarks of an inactive conformation, which includes a mostly disordered activation loop. Here, we present a 2.4 Å resolution crystal structure of an “extended” version of the Mps1 kinase domain, which shows an ordered activation loop. However, the other structural characteristics of an active kinase are not present. Our structure shows that the Mps1 activation loop can fit to the ATP binding pocket and interferes with ATP, but less so with inhibitors binding, partly explain the potency of various Mps1 inhibitors.

## 1 INTRODUCTION

Cell division is one of the most dynamic processes in cell cycle, and is orchestrated by a series of highly regulated events. To ensure accurate chromosomal segregation, cells have evolved a surveillance mechanism called the spindle assembly checkpoint (SAC). The SAC monitors the attachment of spindle microtubules to kinetochores prior to the anaphase of mitosis. In case of attachment errors, the SAC delays the anaphase until all chromosomes regain the properly attached to microtubules.^1^ A main component of the SAC is the monopolar spindle 1 (Mps1) kinase. Mps1 acts as a master conductor of SAC signaling by phosphorylating various kinetochore components.^2^

**Figure 1:**
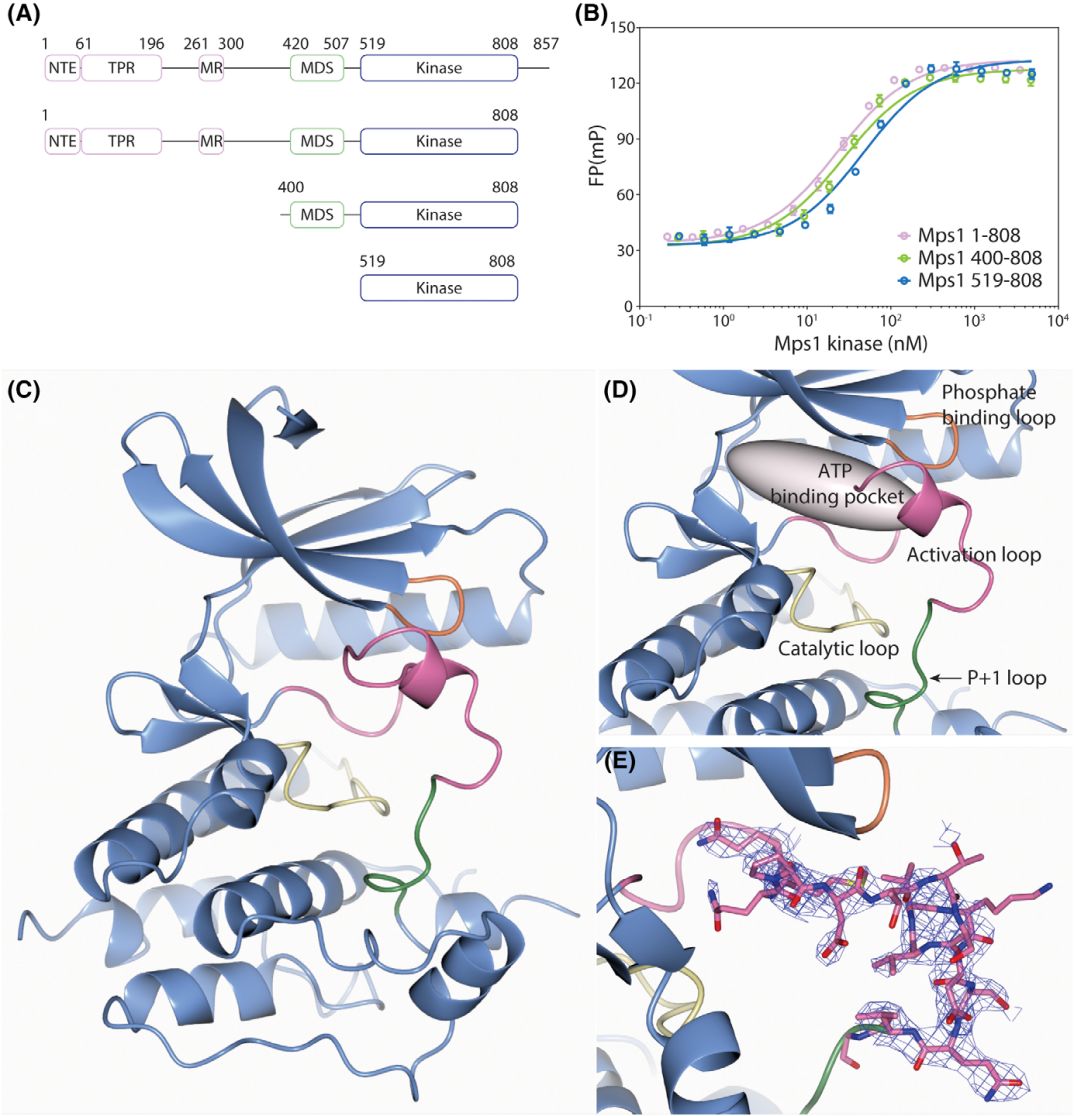
MDS enhances the Mps1 kinase activity. A, Schematic diagram of Mps1 variants. B, FP measurements of turnover of phosphorylation of a Knl1 peptide. Red, green, and blue circles represent the measurements with Mps1^1–808^, Mps1^400–808^, and Mps1^519–808^, respectively. C−E, Cartoon representations of the crystal structure of the Mps1 kinase domain with the ordered activation loop. The activation loop, catalytic loop, phosphate binding loop, and P + 1 loop are highlighted in pink, yellow, orange, and green, respectively. C, Overall structure‐fold of the Mps1 kinase domain. D, ATP binding pocket is depicted as a gray ellipsoid. E, 2mF_o_‐DF_c_ electron density map of the activation loop contoured at 1.0σ

Mps1 kinase consists of a series of N‐terminal functional modules that ensure its localization to kinetochores, the N‐terminal extension (NTE), the tetratricopeptide repeat, and the middle region (Figure 1A); the NTE motif is reported to contribute to the activation of the kinase domain.^3^ The Mps1 degradation signal (MDS) spans across residues 420‐507, mediating the regulation of cellular Mps1 levels.^4^ The Mps1 kinase domain is at the C‐terminal end (519‐808) and has been extensively studied, also as a potential drug target for a cancer therapy.^2^, ^5^ To date, more than 60 crystal structures of the Mps1 kinase domain have been deposited in the protein data bank (PDB). The Mps1 kinase domain adopts the canonical kinase architecture with an N‐terminal lobe connected with a hinge loop to the C‐terminal lobe that contains the catalytic loop, the activation loop, and the P + 1 loop.^5^ Strikingly, all the PDB entries of the Mps1 kinase structures adopt the “inactive conformation” that is defined by several canonical features: (1) a disordered activation loop; (2) a disorientated hydrophobic spine between the Asp‐Phe‐Gly (DFG) and His‐Arg‐Asp (HRD) sequence motifs; and (3) displacement of the αC helix of the N‐terminal lobe, leading to the disruption of the salt bridge between catalytic residues, Lys 553 and Glu 571.^5^ Interestingly, the Mps1 kinase domain co‐crystallized with ATP, also adopts a fully inactive conformation.^6^

In this work, we present the crystal structure of the Mps1 kinase with an ordered activation loop. However, the observed conformation of the loop, which is affected by crystal contacts, blocks access to the ATP binding pocket, and the kinase remains in the inactive conformation. We explore the structure and compare of the Mps1 kinase domain to previously reported structure models and discuss the functional significance of the activation loop.

## 2 MATERIALS AND METHODS

### 2.1 Protein production

The Mps1 constructs, residues 1‐808, 400‐808, and 519‐808, were cloned into the pFastBac‐HT B vector for insect cell expression. An N‐terminal 6xHis tag was introduced, followed by the PreScission Protease site. The sequence verified vector was transformed into DH10Bac cells for bacmid preparation. Recombinant baculovirus was generated following the manufacturer's instructions (Invitrogen). *Spodoptera frugiperda* (Sf9) insect cells were infected with the baculovirus and allowed to grow for 72 hours at 27°C. Cells were harvested by centrifugation at 4000*g* for 20 minutes and re‐suspended in 50 mL of 20 mM KP_i_, pH 7.5, 150 mM KCl, and 1 mM TCEP (buffer A) supplemented with 10 mM imidazole and one tablet of Pierce Protease Inhibitor Tablets EDTA‐free (Thermo Fisher Scientific). Samples were stored at −20°C before proceeding to purification. The re‐suspended cells were lysed by sonication for 1 minute at 50% amplitude in a Qsonica Sonicator Q700 (Fisher Scientific). Following centrifugation at 21 000*g* for 20 minutes at 4°C, the supernatant was incubated with Ni^2+^ charged Chelating Sepharose Fast Flow resin (GE Healthcare) for 30 minutes at 4°C. After extensive washing in buffer A supplemented with 500 mM KCl and 5 mM imidazole, the protein was eluted in 15 mL of 20 mM KP_i_, pH 7.5, 50 mM KCl, and 1 mM TCEP (buffer B), supplemented with 300 mM imidazole. The eluent containing Mps1 was subsequently diluted twofold in buffer B and loaded on a HiTrap Q HP column (GE Healthcare); Mps1 was located in the flow‐through which was collected, concentrated Amicon Ultra‐15 mL −10 K (Merck) and loaded on a Superdex G75 16/60 HiLoad (GE Healthcare) pre‐equilibrated in 20 mM HEPES/NaOH, pH 7.5, 50 mM KCl, and 1 mM TCEP. The protein fractions were pooled together and concentrated to ~120 μM (~5.5 mg mL^−1^). The purified protein stored in 50 μL aliquots, flash‐frozen by liquid nitrogen and stored at −80°C.

### 2.2 Crystallization

The purified Mps1^400‐808^ was crystallized together with ATP, using the sitting drop vapor diffusion method in MRC 2‐Well Crystallization Plates (Hampton Research), with standard screening procedures.^7^ The protein solution at ~120 μM was preincubated with 10 mM of ATP, and 0.1 μL of this solution was mixed with 0.1 μL of reservoir solution and equilibrated against a 50 μL reservoir. Crystals were obtained in the Protein Complex Suite (Qiagen) condition 60%:20% (wt/vol) polyethylene glycol (PEG) 8000, 200 mM ammonium sulfate and 100 mM MES/NaOH, pH 6.5. Crystals appeared at 18°C within 2 weeks. Crystals were briefly transferred to a cryo‐protectant solution containing the reservoir solution and 30% (wt/vol) glycerol and vitrified by dipping in liquid nitrogen.

### 2.3 Data collection and structure refinement

X‐ray data were collected, processed, and analyzed in a fully automated fashion at MASSIF‐1 on beamline ID30A‐1 at the European synchrotron radiation facility (ESRF).^8^ The starting phases were obtained by molecular replacement using PHASER^9^ with an available Mps1 kinase structure (PDB code: 3HMN^6^) as the search model. The models were built and refined using iterative cycles of COOT,^9^ REFMAC,^9^ and PDB‐REDO.^10^ The quality of the models was evaluated by MolProbity.^11^ Data collection and refinement statistics are presented in Table 1.

**Table 1:** Data collection and refinement statistics

PDB identifier	6GVJ
Data collection	
Wavelength (Å)	0.966
Resolution (Å)	58.3‐2.41 (2.45‐2.41)
Space group	I 2 2 2
Unit cell a, b, c (Å)	70.96, 102.61, 111.12
CC_1/2_	0.995 (0.6192)
*R*_merge_	0.0853 (0.5012)
|*I*/σ*I*|	10.40 (2.21)
Completeness (%)	99.10 (90.44)
Multiplicity	4.55 (4.11)
Unique reflections	15 900
Refinement	
Atoms protein/other	2209/68
B‐factors protein/other (Å^2^)	53/59
*R*_work_/*R*_free_ (%)	20.1/23.3
Bond lengths rmsZ/rmsd (Å)	0.334/0.0057
Bond angles rmsZ/rmsd (^o^)	0.543/1.0185
Ramachandran preferred/outliers (%)	97.8/0.0
Rotamers preferred/outliers (%)	92.5/2.0
Clash score (percentile)	100
MolProbity score (percentile)	100

High Resolution shell in parentheses.

### 2.4 Fluorescence‐based Mps1 kinase activity assay

Measurements and data analysis were performed as previously described^12^ with a slight modification: the Mps1 kinase variants were titrated to a reaction mixture containing 20 nM TMR‐Knl1_p_ and 200 nM Bub1/Bub3 complex in 20 mM HEPES/NaOH, pH 7.4, 50 mM KCl, 1 mM ATP, 4 mM MgCl_2_, 1 mM TCEP, and 0.05% Tween20. The samples were incubated at room temperature for 30 minutes in a 384 well Corning assay plate before measurement.

## 3 RESULTS AND DISCUSSION

To investigate the potential role of the MDS region in the Mps1 catalytic activity, we produced the Mps1 construct encompassing residues 400‐808, Mps1^400‐808^. As shown in Figure 1B, the turnover of phosphorylation of a Knl1 peptide was similar between our extended construct Mps1^400‐808^ (26 ± 3 nM) and Mps1^1‐808^ (22 ± 2 nM), about twofold better than for the kinase domain alone (Mps1^519‐808^; 46 ± 6 nM).

To investigate whether the MDS region is interfering with the kinase domain structurally, we then crystallized Mps1^400‐808^ in the presence of ATP. The crystal structure was determined by molecular replacement, at 2.4 Å resolution (Table 1 and Figure 1C−E). However, the electron density did not show any likely placement for the MDS region, suggesting it is either very flexible or degraded during crystallization. Although there is room in the solvent areas of the crystal to accommodate the MDS region, the last visible residue toward the N‐terminus was Asn 515. Notably, neither ATP nor its hydrolysis product ADP was observed in the ATP binding pocket. To our surprise however, while inspecting the structure we noticed that unambiguous density was present for residues 670‐685, the activation loop of the Mps1 kinase (Figure 1E). The activation loop has a random coil structure except residues 677‐680 that form a single helical turn. It should be noted that the observed conformation of the activation loop is stabilized by crystallographic contacts. The side chain of Asn 669 makes a hydrogen bond to the carbonyl oxygen of Gln 736 and backbone nitrogen atom of Ile 738 of a symmetry copy of the molecule. Curiously, while this crystal form is very similar to other crystals we obtained, it is the only one with a clearly ordered activation loop, presumably due to slight differences in crystal packing.

**Figure 2:**
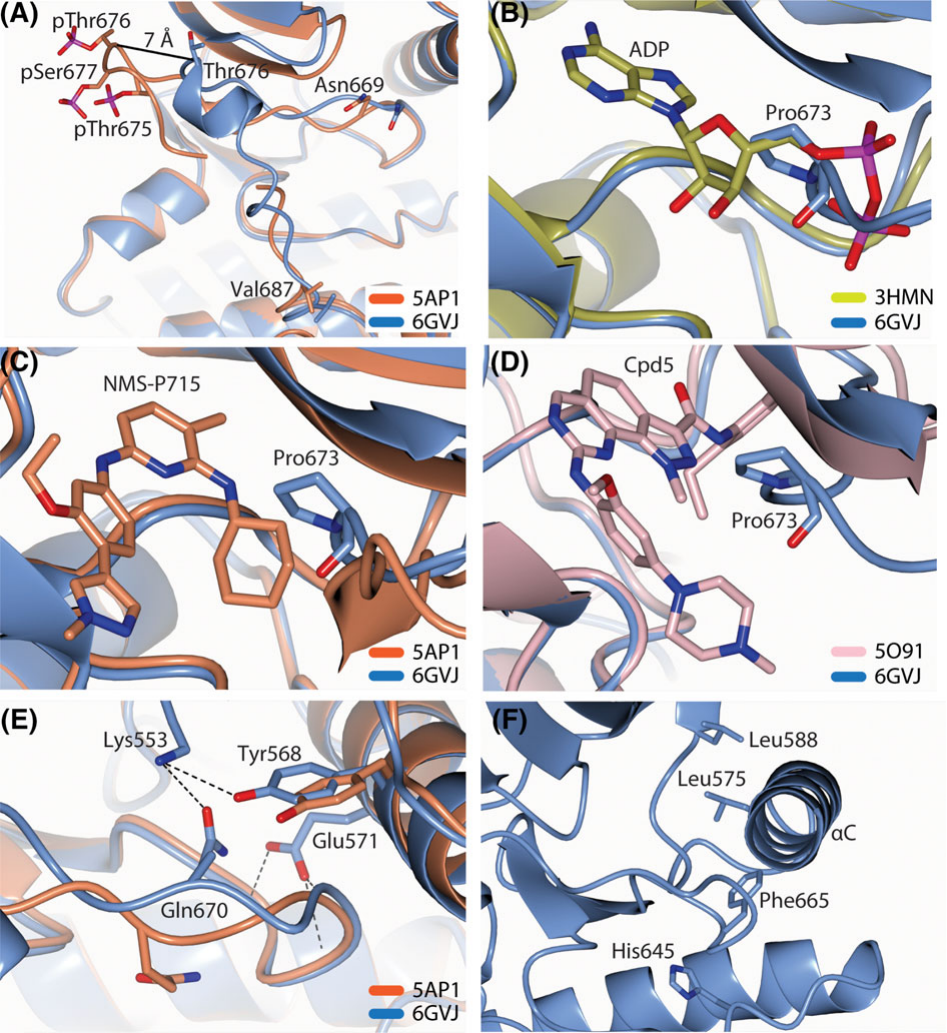
Structure and comparison analysis of the Mps1 kinase domain. The NMS‐P715 bound (5AP1^13^), ADP bound (3HMN^6^), Cpd5 bound (5O91^12^), and current structures (6GVJ) are represented as orange, gold, pink, and blue, respectively. Compounds and annotated residues are depicted as sticks. A, Activation loops with and without phosphorylation. The distance between the positions of the Thr 676 backbone in the two loops is represented as a solid line. B−D, The activation loop severely clashes with ADP and moderately with NMS‐P715 and Cpd5. E and F, Characteristics of inactive conformation of the Mps1 kinase. E, The salt bridge between Lys 553 and Glu 571 is disrupted. Instead, they make hydrogen bonds (black dotted lines) with the residues of the activation loop. F, The side chains building hydrophobic R‐spine of the Mps1 kinase are not aligned

An ordered activation loop is one of the indicators of an active conformation of a kinase. The best‐ordered activation loop in Mps1 structures, is found in 5AP1,^13^ which is in complex with an inhibitor, and also has residues Thr 675, Thr 676, and Ser 677 phosphorylated. The activation loop has fairly clear electron density for the main chain of all residues but 682‐683, which are not modeled and not visible in the electron density. Comparison with our structure shows that the orientation of the activation loop, albeit it occupies a similar space in both structures, diverges already at residue Asn 669 (Figure 2A) and converges back at residue Val 687. The position of the Thr 676 backbone is approximately 7 Å apart between the two loops. A phosphorylated activation loop, as in 5AP1, is a hallmark of activated canonical RD kinases, whereas a phosphoryl group promotes ordering and engaging in electrostatic interactions with the Arg (R) preceding the catalytic Asp (D) residue. Such interactions are not present either in the phosphorylated 5AP1 structure or in ours. Previous reports suggest that Mps1 is not a canonical RD kinase and instead the basic residues located in the C‐terminal lobe, including Lys 706, Lys 708, and Lys 710 have been proposed to interact with the phosphoryl group on Thr 676 to mediate arrangement of the activation loop.^14^ The distances between Thr 676 and those basic residues are larger than 30 Å in the phosphorylated loop structure, as well as in ours. It thus remains to be determined how the phosphorylation of the activation loop becomes an initiating step for Mps1 activation.

Another hallmark of an active kinase conformation is the presence of an ATP molecule. We thus proceeded to compare our structure to the crystal structure of the Mps1 kinase domain bound to an ATP (PDB code: 3HMN^6^). It should be noted however, that inspection of the electron density of 3HMN in the PDB‐REDO databank, clearly suggests that an ADP and not an ATP molecule is bound to that structure as no density is present for the γ‐phosphate, which would be consistent with ATP turnover to ADP by the active kinase under the conditions described for this structure. We used the corrected 3HMN model, containing ADP, for further comparisons. Superposition of our structure and 3HMN shows that the ordered activation loop in our structure (but not that in 5AP1) would severely clash with the α‐, β‐phosphoryl groups, and the ribose ring of the ADP molecule (Figure 2B). We note that while the activation loop in our structure interferes with ADP (and ATP) binding, it does not interfere as strongly with binding of inhibitors, such as NMS‐P715 in 5AP1 (Figure 2C), or Cpd5 in 5O91 (Figure 2D). This might partially explain the potency of these competitive inhibitors, which displace ATP by interfering with the positioning of the adenine base, but allow potential conformations of the dynamic ensemble of activation loop structures in solution (as the two we likely “freeze out” in 5AP1 and our structure) to still exist.

Proceeding with structure analysis, we looked at additional established characteristics of an active kinase structure. The conserved acidic residue, Glu 571 in the αC helix, does not form a salt bridge with the catalytic Lys 553, which in turn stabilizes the ATP binding^5^; instead, the backbone nitrogen atoms of activation loop residues, Phe 665, Ile 667, and Ala 668 make a hydrogen bonding network with Glu 571 (Figure 2E). Notably, the PEG molecule, which is required for the Mps1 crystallization and is often present in the ATP binding pocket as a “halo” on the catalytic Lys 553,^5^ is not observed in our structure with the ordered activation loop. However, the salt bridge formation is disrupted in a manner similar to other inactive conformations. Interestingly, however, the side chain of the Gln 670 residue is rotated by approximately 90° and its oxygen atom is able to make a weak hydrogen bond to Lys 553 (Figure 2E). In addition, the side chain of the Tyr 568 also moves toward the Lys 553 and positions the polar hydroxyl group of the tyrosyl ring close to the lysyl amine (less than 4 Å). Another characteristic feature of an active kinase conformation is the assembly of the “hydrophobic R‐spine,” composed in Mps1 by the side chains of four hydrophobic residues: Leu 588 residue of the β4 strand, Leu 575 residue of the αC helix, Phe 665 residue of the DFG (Asp‐Phe‐Gly) motif, and His 645 residue of the non‐canonical HSD (His‐Ser‐Asp) motif.^15^ In active kinases, these side chains of the hydrophobic residues are aligned and build the “spine” of the kinase‐fold. In the current structure, the hydrophobic R‐spine is disassembled (Figure 2F), also suggesting that our structure is in an inactive conformation.

In summary, we have characterized the Mps1 kinase with the MDS region, showing that inclusion of the MDS restores the full activity of Mps1 toward a Knl1 peptide. Furthermore, the crystal structure of the Mps1^400‐808^ construct revealed an ordered conformation of the activation loop; however the kinase remains in an inactive conformation. Given that the structure of Mps1 in complex with ATP is almost certainly a complex with ADP, and the structure of the triple‐phosphorylated activation loop is not fully ordered and restricted by crystal contacts like the unphosphorylated activation loop in our structure, the structural basis of the Mps1 activation mechanism remains an issue for further investigation.

## Supporting information


**Appendix S1:** Full wwPDB X‐ray Structure Validation Report

## References

[1] Sacristan C , Kops GJPL . Joined at the hip: kinetochores, microtubules, and spindle assembly checkpoint signaling. Trends Cell Biol. 2015;25:21‐28. 10.1016/j.tcb.2014.08.006. 25220181

[2] Pachis ST , Kops GJPL . Leader of the SAC: molecular mechanisms of Mps1/TTK regulation in mitosis. Open Biol. 2018;8:180109 10.1098/rsob.180109. 30111590 PMC6119859

[3] Combes G , Barysz H , Garand C , et al. Mps1 phosphorylates its N‐terminal extension to relieve autoinhibition and activate the spindle assembly checkpoint. Curr Biol. 2018;28:872‐883.e5. 10.1016/j.cub.2018.02.002. 29502948 PMC5863767

[4] Pike AN , Fisk HA . Centriole assembly and the role of Mps1: defensible or dispensable? Cell Div. 2011;6:9 10.1186/1747-1028-6-9. 21492451 PMC3094359

[5] Liu X , Winey M . The MPS1 family of protein kinases. Annu Rev Biochem. 2012;81:561‐585. 22482908 10.1146/annurev-biochem-061611-090435 PMC4026297

[6] Chu ML , Lang Z , Chavas LM , et al. Biophysical and X‐ray crystallographic analysis of Mps1 kinase inhibitor complexes. Biochemistry. 2010;49:1689‐1701. 20099905 10.1021/bi901970c

[7] Newman J , Egan D , Walter TS , et al. Towards rationalization of crystallization screening for small‐ to medium‐sized academic laboratories: the PACT/JCSG+ strategy. Acta Crystallogr D Biol Crystallogr. 2005;61:1426‐1431. 16204897 10.1107/S0907444905024984

[8] Svensson O , Gilski M , Nurizzo D , Bowler MW . Multi‐position data collection and dynamic beam sizing: recent improvements to the automatic data‐collection algorithms on MASSIF‐1. Acta Crystallogr Sect D Struct Biol. 2018;74:433‐440. 10.1107/S2059798318003728. 29717714 PMC5930350

[9] Winn MD , Ballard CC , Cowtan KD , et al. Overview of the CCP4 suite and current developments. Acta Crystallogr D Biol Crystallogr. 2011;67:235‐242. 21460441 10.1107/S0907444910045749 PMC3069738

[10] Joosten RP , Long F , Murshudov GN , Perrakis A . The PDB_REDO server for macromolecular structure model optimization. IUCrJ. 2014;1:213‐220. 10.1107/S2052252514009324 PMC4107921 25075342

[11] Davis IW , Leaver‐Fay A , Chen VB , et al. MolProbity: all‐atom contacts and structure validation for proteins and nucleic acids. Nucleic Acids Res. 2007;35:W375‐W383. 17452350 10.1093/nar/gkm216 PMC1933162

[12] Hiruma Y , Koch A , Hazraty N , et al. Understanding inhibitor resistance in Mps1 kinase through novel biophysical assays and structures. J Biol Chem. 2017;292:14496‐14504. 10.1074/jbc.M117.783555. 28726638 PMC5582842

[13] Gurden MD , Westwood IM , Faisal A , et al. Naturally occurring mutations in the MPS1 gene predispose cells to kinase inhibitor drug resistance. Cancer Res. 2015;75:3340‐3354. 26202014 10.1158/0008-5472.CAN-14-3272

[14] Kang J , Chen Y , Zhao Y , Yu H . Autophosphorylation‐dependent activation of human Mps1 is required for the spindle checkpoint. Proc Natl Acad Sci U S A. 2007;104:20232‐20237. 18083840 10.1073/pnas.0710519105 PMC2154414

[15] Bayliss R , Fry A , Haq T , Yeoh S . On the molecular mechanisms of mitotic kinase activation. Open Biol. 2012;2:120136 10.1098/rsob.120136. 23226601 PMC3513839

